# Pre‐analytical optimization of cell‐free DNA and extracellular vesicle‐derived DNA for mutation detection in liquid biopsies

**DOI:** 10.1002/1878-0261.70222

**Published:** 2026-02-22

**Authors:** Jonas Dohmen, Lucrezia De Santis, Bret M. Stephens, Vincent Bernard, Jörg C. Kalff, Lara Braun, Phillipp Leyendecker, Johannes Röttgen, Daniel Weissinger, Anirban Maitra, Paola A. Guerrero, Alexander Semaan

**Affiliations:** ^1^ Department of Surgery University of Bonn Germany; ^2^ Sheikh Ahmed Pancreatic Cancer Research Center The University of Texas MD Anderson Cancer Center Houston TX USA; ^3^ Department of General and Visceral Surgery Charité Campus Benjamin Franklin Berlin Germany

**Keywords:** cell‐free DNA (cfDNA), digital droplet PCR (ddPCR), extracellular vesicle‐derived DNA (evDNA), liquid biopsy, plasma biomarkers, pre‐analytical variables

## Abstract

Liquid biopsies enable noninvasive tumor profiling and longitudinal disease monitoring. Their analytical performance is strongly influenced by pre‐analytical factors, yet direct comparisons between cell‐free DNA (cfDNA) and extracellular vesicle‐derived DNA (evDNA) remain scarce. We prospectively evaluated four pre‐analytical variables: processing delay, storage temperature, tube type, and plasma input volume, on cfDNA and evDNA from cancer patient plasma (*n* = 244) using ddPCR, Qubit, and TapeStation. Key findings were validated in archived plasma samples (*n* = 723). In the prospective cohort, cfDNA concentrations increased after 24 h and evDNA after 48 h at room temperature, while retrospective analysis revealed earlier changes (cfDNA: 6 h; evDNA: 24 h). Storage conditions influenced both analytes, as short‐term refrigeration (4 °C) better preserved DNA quality than −80 °C freezing, while extracted DNA remained stable at −80 °C. Acid citrate dextrose (ACD) and K_2_EDTA tubes performed similarly under prompt processing. Higher plasma volumes improved evDNA, but not cfDNA, for mutation detection. evDNA demonstrates greater resilience than cfDNA under suboptimal conditions. Standardized workflows and prompt processing are essential to ensure reliable mutation detection in clinical liquid biopsy applications.

## Graphical abstract

Pre‐analytical handling critically determines liquid biopsy performance. This study defines practical best‐practice conditions for cell‐free DNA (cfDNA) and extracellular vesicle–derived DNA (evDNA), showing how processing time, storage conditions, tube type, and plasma input volume affect DNA integrity and mutation detection. The findings highlight evDNA as the more resilient analyte and provide guidance for standardized and reliable molecular cancer diagnostics.

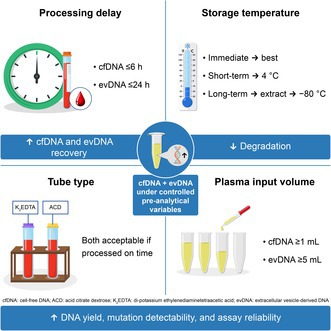

**Abbreviations**
ACD: acid citrate dextrose
AMP: Association for Molecular Pathology
ASCO: American Society of Clinical Oncology
Bp: base pairs
CAP: College of American Pathologists
cfDNA: cell‐free DNA
CRC: colorectal carcinoma
CTC: circulating tumor cell
ddPCR: digital droplet polymerase chain reaction
ESMO: European Society for Medical Oncology
EV: extracellular vesicles
evDNA: extracellular vesicle‐derived DNA
FDA: Food and Drug Administration
ISO: International Standards Organization
K_2_EDTA: di‐potassium ethylenediaminetetraacetic acid
*KRAS*
: Kirsten rat sarcoma virus oncogene homolog
*KRAS* ^mut^
: mutant *KRAS* (determined by droplet digital PCR)
LOD: limit of detection
NCI: National Cancer Institute
NGS: next generation sequencing
PBS: phosphate‐buffered saline solution
PCR: polymerase chain reaction
PDAC: pancreatic ductal adenocarcinoma
RT: room temperature

## 1 Introduction

Liquid biopsies have revolutionized translational cancer research, enabling real‐time, noninvasive monitoring of tumor genetics and treatment responses [1, 2, 3, 4]. This approach provides clinicians with predictive and prognostic insights, supporting treatment decisions and patient stratification. Numerous studies have demonstrated the clinical utility of liquid biopsies in early cancer detection, mutational profiling, resistance monitoring, and disease surveillance [5, 6, 7, 8, 9, 10, 11, 12, 13, 14, 15]. By enabling dynamic, minimally invasive tumor characterization, liquid biopsies hold immense potential to improve patient outcomes [1].

Liquid biopsies primarily analyze three circulating compartments: cell‐free DNA (cfDNA), circulating tumor cells (CTCs), and extracellular vesicles (EV) such as exosomes, which have emerged as a complementary platform for tumor‐derived DNA [16]. A major challenge lies in the reliable detection of rare tumor‐derived mutations, given the low fraction of tumor DNA in circulation. This challenge is particularly evident in early‐stage cancers or in patients undergoing therapies [17, 18, 19]. cfDNA, released predominantly from apoptotic and necrotic tumor cells, is highly susceptible to degradation, making timely and standardized sample handling critical for accurate analysis [20, 21, 22, 23, 24, 25]. Studies suggest that shorter cfDNA fragments are more frequently detected in blood samples processed immediately after collection, compared to those subjected to processing delays [20, 21, 26].

In contrast, EV, actively secreted by living cells, offer several biological and technical advantages. DNA derived from extracellular vesicles (evDNA) is present in high abundance in bodily fluids, making it readily accessible for analysis. Unlike cfDNA, EV are released by viable cells and may therefore better reflect the current tumor biology, potentially enabling higher sensitivity and specificity in mutation detection. Their lipid bilayer membrane confers protection against enzymatic degradation, enhancing the stability of their nucleic acid and protein cargo during storage and processing [16, 27, 28]. In addition, EV are implicated in intercellular communication and play a critical role in the formation of the premetastatic niche, thereby promoting tumor progression and metastasis [29, 30]. However, several challenges remain. The heterogeneity of EVs and overlapping size ranges of different vesicle populations complicate their classification and functional characterization [31]. Isolation methods must achieve high efficiency and purity, as co‐isolated contaminants can compromise downstream analyses, which greatly increase processing time [28]. Moreover, isolation protocols vary widely across studies, highlighting the need for standardization [32]. Despite these challenges, EV represent a promising and complementary analyte in liquid biopsies, offering the potential to detect tumor‐specific molecular alterations with greater stability and biological relevance than cfDNA [31, 32, 33].

Standardization of pre‐analytical variables—including processing time, storage temperature, collection tube type and input volume—is essential to ensure reproducibility, comparability, and clinical utility of liquid biopsy assays [34, 35, 36].

It is generally advised that blood samples for cfDNA analysis be processed as quickly as possible, as prolonged storage can lead to degradation of nucleic acids [37, 38]. However, logistical constraints often necessitate temporary storage prior to plasma processing.

Despite extensive research, the precise effects of short‐time delayed processing on cfDNA quality remain inconclusive [22, 39]. While some studies suggest that cfDNA remains stable up to 24 h [40], others recommend processing within 2–4 h at room temperature to minimize leukocyte lysis and wild‐type DNA contamination [38, 41]. In contrast, EVs, including exosomes, demonstrate greater stability in circulation. Several studies report that EV‐associated DNA can remain stable for up to 48 h or even a few days at room temperature [42, 43].

Storing cfDNA and evDNA at −80 °C is considered best practice for long‐term preservation to minimize degradation and maintain sample quality [34, 38, 43, 44]. For short‐term storage, 4 °C is generally preferred over room temperature, with some studies supporting cfDNA stability for at least 72 h at 4 °C [45, 46], while others recommend limiting storage to 8 h [47]. However, already extracted cfDNA can be reliably used for up to 9 months when stored at −20 °C or − 80 °C [48]. For evDNA, short‐term storage at 4 °C has also proven to be a viable option, with stability reported for up to 1 week [43].

The optimal choice of blood collection tubes for liquid biopsy applications remains under evaluation [49]. While specialized, cell‐stabilizing tubes (e.g., Streck BCT) can preserve cfDNA quality for extended periods [36, 50, 51], standard EDTA tubes are most cost‐effective, accessible and perform equally well when samples processed promptly [40, 52, 53]. As a result, EDTA tubes are widely regarded as the current gold standard for liquid biopsy workflows [54]. Among non‐stabilized collection tubes, both citrate‐based (e.g., ACD = Acid citrate dextrose) and EDTA‐based tubes have demonstrated comparable performance, whereas heparin tubes are generally not recommended due to their known interference with downstream PCR‐based applications [55, 56].

Researchers relying on biobank‐derived plasma samples often face limitations in cfDNA quantity, which may impact downstream analysis. As tumor‐derived cfDNA is typically present in only trace amounts, larger plasma volumes are generally required to ensure adequate DNA recovery for reliable mutation detection [57, 58]. Increasing plasma input volume has been shown to enhance cfDNA yield and improve the probability of detecting tumor‐specific mutations [59]. Some researchers recommend collecting 2 × 10 mL of blood per patient [53]. However, no standardized guidelines for plasma volume exist, and comprehensive studies assessing optimal collection volumes remain limited [37].

To address these ongoing uncertainties, we systematically analyzed the impact of key pre‐analytical variables, including processing delay, storage temperature, tube type, and plasma input volume, on cfDNA and evDNA quality and mutation detection performance in a large cohort of patients with pancreatic ductal adenocarcinoma (PDAC) or colorectal carcinoma (CRC), using *KRAS* mutation (*KRAS* ^mut^) detection as a representative molecular readout within a standardized digital droplet PCR (ddPCR) workflow.

## 2 Materials and methods

### 2.1 Cohorts

To systematically assess the impact of pre‐analytical variables on the yield and mutation detection performance of cfDNA and evDNA, we analyzed patient blood samples under controlled variations in processing time, storage temperature, collection tube type, and plasma input volume.

**Fig. 1:**
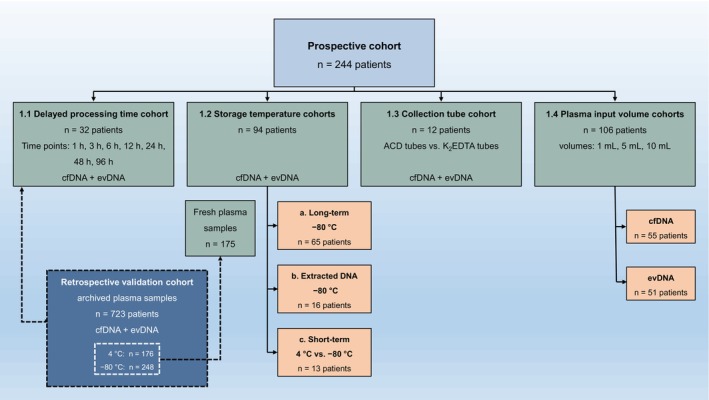
Study design and sample allocation across pre‐analytical subcohorts. The flow chart summarizes the distribution of all 244 prospective plasma samples across four experimental subcohorts (delayed processing time, storage temperature, collection tube type, and plasma input volume). Each patient contributed to one specific subgroup only; no overlap occurred between subcohorts. The retrospective validation dataset (*n* = 723) is shown separately at the bottom.

A total of 244 plasma samples from 244 individual patients were included in the prospective analyses. Each patient contributed to one specific experimental subgroup; no samples were reused across different pre‐analytical conditions. Study design and sample allocation are summarized in Fig. 1.

#### 2.1.1 Delayed processing time cohort (*n* = 32, Table S1)

This cohort evaluated the effects of delayed whole‐blood processing on cfDNA and evDNA. Blood was collected in ACD tubes (8.5 mL Vacutainer ACD Tube; BD Biosciences, San Jose, CA, USA), and plasma was separated at predefined time points: 1, 3, 6, 12, 24, 48, and 96 h post‐venipuncture at room temperature (19 °C–25 °C). To validate these findings, we retrospectively analyzed archived patient samples in our institutional liquid biopsy database (*n* = 723; 641 PDAC, 82 CRC, Table S2).

#### 2.1.2 Storage temperature cohorts (*n* = 94)

- Long‐term plasma cryopreservation at −80 °C: This cohort (*n* = 65, Table S3) evaluated the impact of plasma cryopreservation at −80 °C for 2 weeks versus immediate processing on cfDNA and evDNA yield.
- Storage of extracted DNA at −80 °C: Extracted cfDNA and evDNA samples from patients (*n* = 16) were analyzed immediately after extraction and reanalyzed after storage at −80 °C for 2 weeks.
- Short‐term plasma storage: immediate vs. 4 °C vs. −80 °C overnight: Bood samples (*n* = 13) were collected in ACD tubes, processed within 3 hours, and plasma was divided into three 6 mL aliquots: one was processed immediately (reference), one stored at 4 °C overnight, and one stored overnight at −80 °C.

To validate these findings on a larger scale, we compared prospectively collected fresh plasma samples (*n* = 175) with archived plasma samples stored at 4 °C (*n* = 176) and − 80 °C (*n* = 248) from our institutional liquid biopsy database.

#### 2.1.3 Collection tube cohort (*n* = 12, Table S4)

This cohort compared cfDNA and evDNA concentrations from blood collected in ACD tubes versus di‐potassium ethylenediaminetetraacetic acid (K_2_EDTA) tubes (10 mL Vacutainer K_2_EDTA Plastic Tube; BD Biosciences, USA). Each patient provided three tubes of each type, and plasma from both conditions was processed using the standardized extraction workflow (see below).

#### 2.1.4 Plasma input volume cohorts (in total *n* = 106)

These cohorts investigated the impact of varying plasma input volumes (1, 5, 10 mL) on cfDNA (*n* = 55, Table S5) and evDNA (*n* = 51, Table S6) yield.

### 2.2 Patient samples and plasma preparation

Each patient provided approximately 25 mL of blood, processed according to cohort‐specific protocols. For most cohorts, three ACD tubes of whole blood were collected and processed within 3 h of venipuncture under standardized protocols. In the collection tube cohort, six tubes were collected per patient (three ACD tubes and three K_2_EDTA).

Whole blood was centrifuged at 2500× ***g*** for 10 min at 22 °C (Thermo Sorvall Legend RT; Thermo Fisher Scientific, Waltham, MA, USA). Plasma was further centrifuged at 1000× ***g*** for 5 min at 4 °C, and the supernatant was spun at 5000× ***g*** for 10 min. After each cycle, the supernatant was decanted in a new conical tube to remove platelets and cellular debris from the plasma sample. EV were isolated using a serial ultracentrifugation protocol at 4 °C as described previously [60]. The plasma was diluted in ice‐cold phosphate‐buffered saline solution (Corning PBS without calcium and magnesium; Thermo Fisher Scientific, Corning, NY, USA) balanced in ultracentrifuge tubes (Ultra Centrifuge Bottle PC 100 mL; Thermo Fisher Scientific, USA) and spun at 1 54 000× ***g*** overnight in an Ultracentrifuge (Thermo Sorvall WX Floor Ultra Centrifuge; Thermo Fisher Scientific, USA). The resulting EV pellet was washed with ice‐cold PBS and ultracentrifuged for 2 additional hours before resuspension in 200–600 μL of ice‐cold PBS, depending on the plasma input volume.

### 2.3 DNA isolation and processing

cfDNA and evDNA were extracted using QIAamp Circulating Nucleic Acid Kits (Qiagen, Germantown, MD, USA) on a QIAvac 24 Plus vacuum manifold. Standard extractions were performed from 1 mL of plasma for cfDNA and 200 μL of EV suspension for evDNA. In the plasma input volume cohort, extractions were conducted from 1, 5, and 10 mL of plasma. The QIAamp extraction kits were used according to the manufacturer's instructions with two modifications:

1. DNA was eluted twice from the Qiagen column using 50 μL molecular‐grade water (Corning™ Molecular Biology Grade Water; Thermo Fisher Scientific, USA) to maximize yield. The resulting eluate underwent isopropanol‐precipitation step to purify the sample by adding 2 μL linear acrylamide (Invitrogen Linear Acrylamide; Thermo Fisher Scientific, USA), 10 μL sodium acetate (Sodium Acetate 3 m Buffer Solution; Sigma‐Aldrich, St. Louis, MO, USA), and 100 μL of molecular grade isopropanol (Thermo Fisher Scientific, USA). The DNA solution was cryopreserved (−80 °C) until downstream analysis.
2. The isopropyl‐DNA solution was centrifuged at 15 000× ***g*** for 20 min at 4 °C. The resulting pellet was dried in a thermomixer (Eppendorf ThermoMixer C; Thermo Fisher Scientific, USA) at 37 °C for 10 min. The DNA was diluted to a final volume of 12 μL of molecular grade water.

This standardized workflow was applied across all patient samples to ensure consistency in cfDNA and evDNA isolation.

All samples were analyzed by ddPCR prior to cryopreservation at −80 °C for long‐term storage, with the exception of those in the storage temperature cohort (see Results section for details). ddPCR was selected for its high sensitivity and low limit of detection (LOD) (0.01%–0.05%), outperforming next‐generation sequencing (NGS) in identifying rare mutant alleles in low‐burden samples [61, 62, 63, 64].

### 2.4 Quantification of circulating KRAS in cfDNA and evDNA

*KRAS* mutations in cfDNA and evDNA were detected using the *KRAS* G12/G13 Screening Kit (Bio‐Rad Laboratories, Hercules, CA, USA), which targets seven actionable mutations in codon 12 and 13 [1, 65, 66]. Each reaction contained 2 μL of cfDNA or evDNA, 6 μL molecular‐grade water and 12 μL Master Mix (containing FAM and HEX fluorescent probes). The samples were measured using the QX200 Droplet Digital PCR System (Bio‐Rad Laboratories, USA). Data analysis was performed with QuantaSoft V1.6.6.0320 software. According to Bio‐Rad's best practice guidelines for rare mutation detection, a minimum of two positive droplets was required for mutation detection. Each ddPCR run included a *Panc1* cell line (positive control), wild‐type DNA (negative control), and no‐template molecular‐grade water (contamination control). The total number of amplifiable *KRAS* target molecules (mutant + wild‐type) per reaction was estimated by QuantaSoft using a Poisson distribution model based on droplet counts for the FAM (mutant) and HEX (wild‐type) probes [52, 67]. These values were normalized to plasma input volume and are reported as amplifiable genome equivalents (copies/μL plasma). DNA mass concentrations (ng/μL) represent ddPCR‐derived estimates calculated from amplifiable genome equivalents using QuantaSoft's default conversion model. Input efficiency was determined by dividing the total number of accepted droplets per reaction by the expected droplet count (20 000 per reaction). Reactions with fewer than 50% of the expected droplets were excluded from analysis. To illustrate stage‐related differences in circulating mutation abundance, *KRAS* ^mut^ detection levels were additionally assessed in cfDNA from PDAC patients. Tumor stage was classified according to the UICC (Stages I/II = early, Stages III/IV = late). ddPCR‐derived allele frequencies were compared between groups using the Mann–Whitney test. Results are shown in Fig. S1, demonstrating higher *KRAS* ^mut^ abundance in late‐stage compared with early‐stage disease (*P* < 0.0001).

### 2.5 DNA quantification methods

- Qubit Fluorometric Assay (Qubit 2.0 Fluorometer; Invitrogen, Carlsbad, CA, USA): 1 μL of DNA was used with the Qubit dsDNA HS Assay Kit (Life technologies, Grand Island, NY, USA) following the manufacturer's instructions.
- TapeStation Bioanalyzer (TapeStation 2200; Agilent, Santa Clara, CA, USA): 2 μL of DNA were analyzed with the TapeStation D1000 HS Assay (Agilent Technologies, Santa Clara, CA, USA), following the manufacturer's instructions.

### 2.6 Statistical analysis

All statistical analyses and figure generation were performed using GraphPad Prism version 10 (Dotmatics Inc, San Diego, CA, USA). Data distribution was assessed for normality or log‐normality using the Shapiro–Wilk test or the D'Agostino–Pearson omnibus test, depending on sample size. With the exception of the retrospective plasma storage validation cohort (fresh vs. 4 °C vs. −80 °C), all datasets consisted of paired measurements. For normally or log‐normally distributed data involving more than two groups without missing values, repeated‐measures one‐way ANOVA with Geisser–Greenhouse correction was applied, followed by Dunnett's multiple comparisons test, when comparing experimental groups to a single control, or Tukey's test when all groups were compared. For datasets containing missing values, Prism's mixed‐effects model was applied. For two‐group comparisons, normally or log‐normally distributed paired data were analyzed using the paired *t*‐test. Nonparametric data involving more than two paired groups were analyzed with the Friedman test followed by Dunn's multiple comparisons test. For two‐group comparisons, the Wilcoxon matched‐pairs signed rank test was applied. Correlations between paired measurements were obtained from the ratio paired *t*‐test output (Pearson correlation coefficient *r*). For Fig. S6, which comprised three independent (unpaired) groups, ordinary one‐way ANOVA on log‐transformed data was applied, followed by Tukey's multiple comparisons test. All data are presented as box‐and‐whisker plots (Tukey style) on either a linear or log_10_ scale. Statistical significance is indicated as *P* < 0.05 (*), *P* < 0.01 (**), *P* < 0.001 (***), and *P* < 0.0001 (****). Sensitivity analyses (ROUT, *Q* = 1%) were additionally performed for all smaller cohorts (*n* < 50) to identify potential outliers. Statistical conclusions remained unchanged after outlier exclusion, confirming the robustness of all reported results.

### 2.7 Ethics approval and informed consent

This study was conducted in accordance with the ethical standards of the institutional and/or national research committee and with the Helsinki Declaration and its later amendments. The experiments were undertaken with the understanding and written consent of each subject. Patients were recruited at MD Anderson Cancer Center, and blood samples were collected between 2016 and 2020. All participants provided written informed consent prior to blood sample collection and molecular analysis following approval by the institutional review board (IRB approvals: PA14‐0867, PA11‐0670, PA14‐0552 and Lab10‐098). Patient data were anonymized prior to analysis to ensure confidentiality, and no identifiable personal information was used or disclosed. The study adhered to all relevant guidelines and regulations governing research involving human participants (Table 1).

**Table 1:** Overview of experimental cohorts, sample sizes, pre‐analytical variables, and analytical readouts. This table summarizes all pre‐analytical variables evaluated in the study, the corresponding sample sizes, analytes tested, and analytical techniques used.

Pre‐analytical variable	Cohort (*n*)	Analyte(s)	Readout/technique	Figures/supplement	Comparison
Delayed processing time	*n* = 32	cfDNA, evDNA	ddPCR (concentration); TapeStation (fragment length)	Fig. 2 Fig. S2	Plasma processed at 1 h vs. 3, 6, 12, 24, 48, 96 h
Delayed processing – extended validation	*n* = 723 (archived samples)	cfDNA, evDNA	ddPCR (concentration)	Fig. 3	Plasma processed at 1 h vs. 6, 24, 96 h
Long‐term plasma storage (−80 °C)	*n* = 65	cfDNA, evDNA	ddPCR (concentration, *KRAS* ^*m*ut^ allele frequency, amplifiable genome equivalents); Qubit (concentration); TapeStation (concentration, fragment length)	Fig. 4 Fig. S3	Fresh vs. −80 °C for 2 weeks
Storage of extracted DNA (−80 °C)	*n* = 16	cfDNA, evDNA	ddPCR (concentration, *KRAS* ^*m*ut^ allele frequency, amplifiable genome equivalents)	Fig. S4	Fresh extract vs. −80 °C for 2 weeks
Short‐term plasma storage (overnight)	Prospective *n* = 13	cfDNA, evDNA	ddPCR (concentration, amplifiable genome equivalents)	Fig. S5	Immediate vs. 4 °C vs. −80 °C overnight
Short‐term storage – extended validation	Fresh: *n* = 175 Archived samples: *n* = 424 (4 °C *n* = 176; −80 °C *n* = 248)	cfDNA, evDNA	ddPCR (concentration)	Fig. S6	Fresh vs. 4 °C vs. −80 °C
Collection tube type	*n* = 12	cfDNA, evDNA	ddPCR (concentration)		ACD vs. K_2_EDTA
Plasma input volume	cfDNA: *n* = 55	cfDNA	ddPCR (*KRAS* ^*m*ut^ allele frequency, amplifiable genome equivalents); Qubit (concentration); TapeStation (concentration, fragment length)	Fig. 5	1 mL vs. 5 mL vs. 10 mL
	evDNA: *n* = 51	evDNA	ddPCR (*KRAS* ^*m*ut^ allele frequency, amplifiable genome equivalents); Qubit (concentration); TapeStation (concentration, fragment length)	Fig. 6	1 mL vs. 5 mL vs. 10 mL

## 3 Results

To evaluate the impact of pre‐analytical variables on cfDNA and evDNA quality and mutation detection, we prospectively analyzed patient samples across four defined experimental cohorts: delayed processing time, storage temperature, blood collection tube type, and plasma input volume. These prospective analyses were conducted using controlled protocols, but were limited in sample size due to logistical constraints. To enhance the robustness and generalizability of our findings, we validated key observations, particularly those from the delayed processing and storage temperature cohorts, using a large retrospective dataset from 723 patients, for which processing times and storage conditions were well‐documented in our institutional liquid biopsy database. Together, this dual approach allowed for a comprehensive assessment of pre‐analytical variables affecting the quality and reliability of cfDNA and evDNA analysis in the context of liquid biopsy.

### 3.1 Delayed processing: cfDNA increases earlier than evDNA

**Fig. 2:**
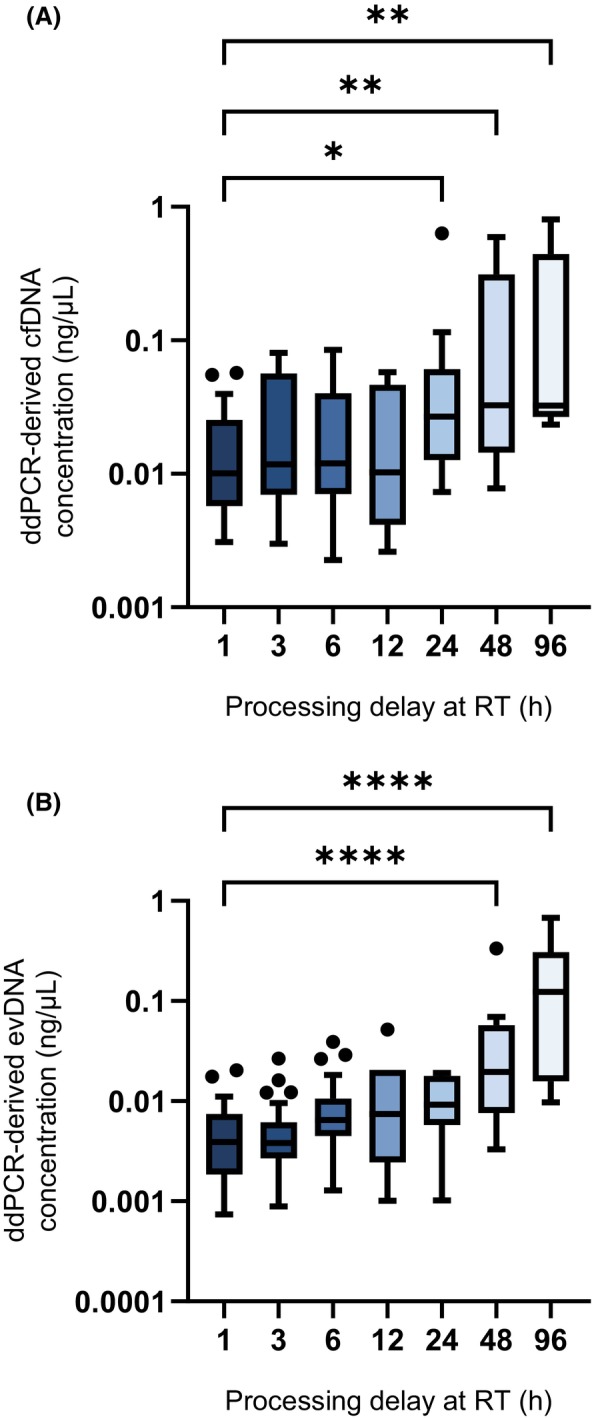
Impact of delayed processing on cfDNA and evDNA yield. (A) Droplet digital PCR (ddPCR)‐derived concentration of cell‐free DNA (cfDNA; ng/μL) in plasma after defined delays in whole‐blood processing at room temperature (RT, 19 °C–25 °C). (B) ddPCR‐derived concentration of extracellular vesicle‐derived DNA (evDNA; ng/μL) after defined delays in whole‐blood processing at room temperature (RT, 19 °C–25 °C). Whole‐blood samples were stored at RT for 1–96 h (h) prior to plasma separation (“processing delay”). Data are shown as box‐and‐whisker plots (Tukey method) on a log_10_ scale. Each data point represents an independent patient sample from the delayed processing time cohort (*n* = 32 patients). All available samples were included, and each patient contributed to one experimental condition only. Gradual color shading (dark to light) indicates increasing processing delay. Statistical significance was assessed using a mixed‐effects model for repeated measures with Geisser–Greenhouse correction, followed by Dunnett's multiple comparisons test, using the 1 h time point as reference. *P < 0.05, **P < 0.01, ****P < 0.0001.

In the prospective delayed processing cohort (*n* = 32), blood samples were processed immediately or after defined delays at room temperature. ddPCR analysis revealed a significant increase in cfDNA concentration after a 24 h delay (Fig. 2A). For evDNA, concentration significantly increased only after 48 h (Fig. 2B).

Fragment size analysis using TapeStation showed that cfDNA and evDNA fragment lengths remained largely unchanged over the 96 h observation period. No statistically significant differences in fragment size were observed for either cfDNA or evDNA (Fig. S2A,B).

**Fig. 3:**
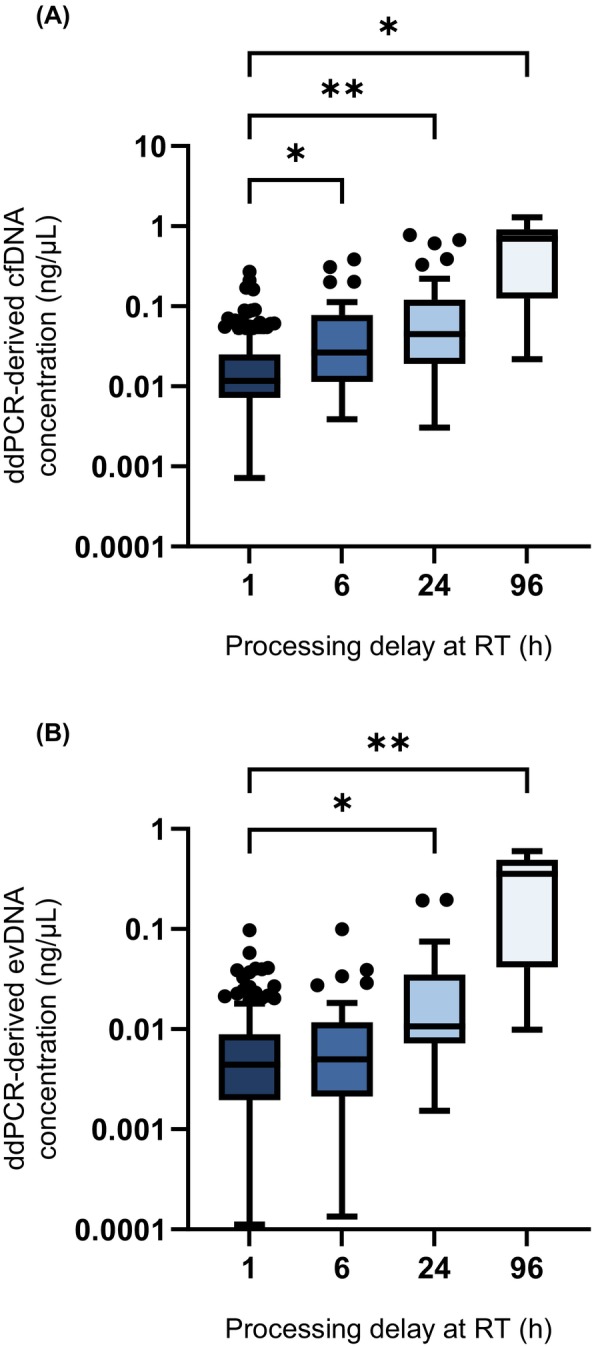
Impact of delayed processing on cfDNA and evDNA yield in an expanded clinical cohort. (A) Droplet digital PCR (ddPCR)‐derived concentration of circulating cell‐free DNA (cfDNA; ng/μL) in plasma after defined delays in whole‐blood processing at room temperature (RT, 19 °C–25 °C). (B) ddPCR‐derived concentration of extracellular vesicle‐derived DNA (evDNA; ng/μL) after defined delays in whole‐blood processing at room temperature (RT, 19 °C–25 °C). Whole‐blood samples were stored at RT for 1–96 h (h) prior to plasma separation (“processing delay”). Data are shown as box‐and‐whisker plots (Tukey method) on a log_10_ scale. Each data point represents an independent patient sample from the expanded retrospective cohort (*n* = 723). All samples available for this analysis were included. Gradual color shading (dark to light) indicates increasing processing delay. Statistical significance was assessed using a mixed‐effects model for repeated measures with Geisser–Greenhouse correction, followed by Dunnett's multiple comparisons test, using the 1 h time point as reference. *P < 0.05, **P < 0.01.

To validate these findings at scale, we performed a retrospective analysis of archived clinical samples (*n* = 723) with available processing times and cfDNA/evDNA concentration data. This analysis revealed the same sequential pattern, but with earlier onset: cfDNA levels increased as early as 6 h postcollection, whereas evDNA concentration rose after 24 h (Fig. 3A,B).

### 3.2 Temperature matters: Plasma freezing impairs cfDNA quality while evDNA remains resilient

**Fig. 4:**
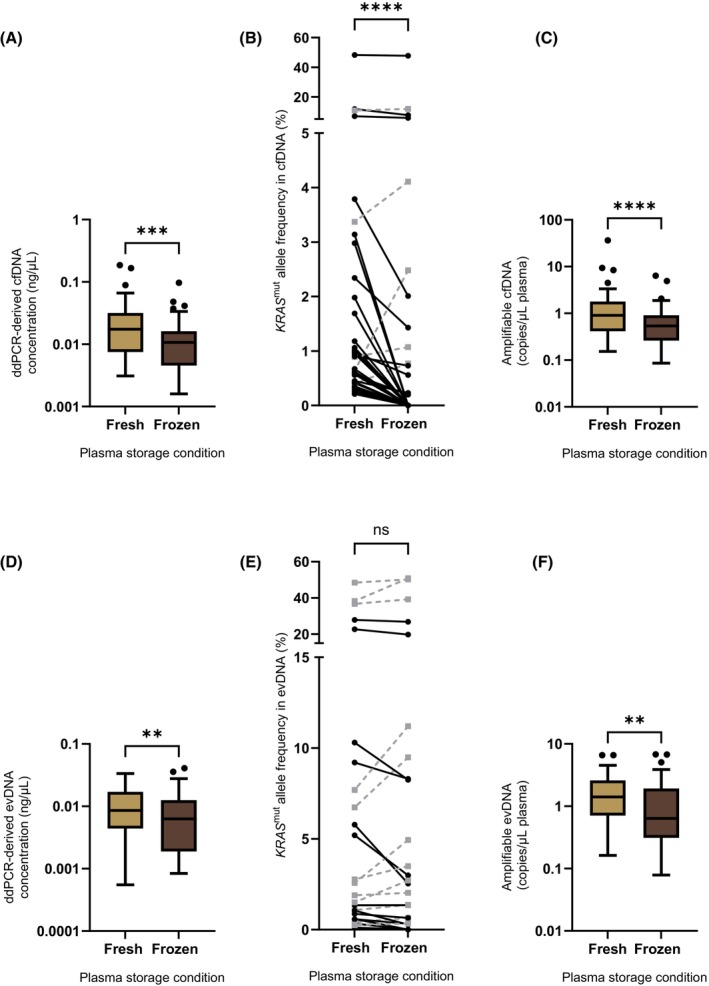
Impact of plasma cryopreservation on cfDNA and evDNA yield and mutation detection. (A) Droplet digital PCR (ddPCR)‐derived concentration of circulating cell‐free DNA (cfDNA; ng/μL) in freshly processed versus cryopreserved (−80 °C) plasma (B) *KRAS*‐mutant (*KRAS*ᵐᵘᵗ) allele frequency (%) in cfDNA under identical conditions; gray dashed lines indicate increases and black solid lines indicate decreases in paired samples. (C) Amplifiable cfDNA (copies/μL plasma) determined by ddPCR. (D) ddPCR‐derived concentration of extracellular vesicle‐derived DNA (evDNA; ng/μL) in freshly processed and cryopreserved plasma (E) *KRA*Sᵐᵘᵗ allele frequency (%) in evDNA; gray dashed lines indicate increases and black solid lines indicate decreases in paired samples. (F) Amplifiable evDNA (copies/μL plasma) determined by ddPCR. Whole‐blood samples were processed either immediately after collection (Fresh) or after cryopreservation at −80 °C for 2 weeks (Frozen). Panels A, C, D and F are displayed on a log_10_ scale; panels B and E on a linear scale. Each data point represents an independent patient sample from the long‐term −80 °C storage temperature cohort (*n* = 65). Statistical significance was assessed using paired *t*‐tests (A, C, D, F) and Wilcoxon matched‐pairs signed‐rank tests (B, E). ns = not significant; **P < 0.01, ***P < 0.001, ****P < 0.0001.

- Long‐term plasma cryopreservation at −80 °C (*n* = 65): Prospective plasma samples were either processed immediately after collection (Fresh) or cryopreserved at −80 °C for 2 weeks (Frozen). ddPCR‐derived cfDNA concentrations (Fig. 4A) and *KRAS* ^mut^ allele frequencies (Fig. 4B) were both significantly lower in cryopreserved compared with freshly processed plasma. Similarly, amplifiable cfDNA genome equivalents decreased upon cryopreservation (Fig. 4C). For evDNA, cryopreservation likewise reduced ddPCR‐derived DNA concentration (Fig. 4D), while *KRAS* ^mut^ allele frequency remained largely unchanged (Fig. 4E). Consistent with the decline in overall DNA yield, amplifiable genome equivalents were also reduced in frozen samples (Fig. 4F). These findings were supported by Qubit and TapeStation analyses, which showed a significant reduction in cfDNA concentration (Fig. S3A,B) and fragment length (Fig. S3C) after plasma freezing. Likewise, evDNA concentration decreased upon cryopreservation (Fig. S3D,E), whereas evDNA fragment length remained unaffected (Fig. S3F).
- Storage of extracted DNA at −80 °C (*n* = 16): Prospective cfDNA and evDNA samples, processed within 3 hours of collection, were analyzed immediately after extraction (Fresh) and again following cryopreservation of purified DNA at −80 °C for 2 weeks (Frozen). ddPCR analysis showed comparable cfDNA and evDNA concentrations, *KRAS*ᵐᵘᵗ allele frequencies, and amplifiable genome equivalents between freshly analyzed and cryopreserved DNA samples (Fig. S4A–F). These results indicate that purified cfDNA and evDNA remain stable during short‐term cryogenic storage after extraction.
- Short‐term plasma storage: immediate vs. 4 °C vs. −80 °C overnight (*n* = 13): Prospective blood samples were processed within 3 hours of collection, and plasma aliquots were either analyzed immediately (Fresh), stored at 4 °C overnight, or cryopreserved at −80 °C overnight (Frozen). ddPCR analysis of cfDNA revealed temperature‐dependent differences in both concentration and amplifiable genome equivalents, with highest levels in freshly processed samples, lower values after storage at 4 °C, and the lowest after −80 °C (Fig. S5A,B). For evDNA, concentrations were comparable between fresh and 4 °C conditions but decreased after −80 °C storage, whereas amplifiable genome equivalents remained largely unchanged across all conditions (Fig. S5C,D).
- To validate these findings, we analyzed archived plasma samples stored overnight at 4 °C (*n* = 176) or at −80 °C (*n* = 248) and compared them with fresh plasma samples from our prospective cohort (*n* = 175). ddPCR analysis revealed a statistically significant decrease in cfDNA concentrations for both storage conditions compared to freshly processed plasma (Fig. S6A). evDNA concentrations also declined after both 4 °C and − 80 °C storage, although no difference was observed between 4 °C and frozen samples in terms of ddPCR‐derived evDNA concentration (Fig. S6B).

Together, these findings underscore the detrimental impact of plasma storage, particularly cryopreservation, on cfDNA quality and mutation detectability, while evDNA demonstrates greater resilience under suboptimal conditions.

### 3.3 Comparable performance of ACD and K_2_ EDTA tubes

In this cohort (*n* = 12), cfDNA and evDNA yields were compared between matched blood samples collected in ACD and K_2_EDTA tubes. Median cfDNA concentrations were highly similar between tube types (ACD: 0.0117 ng/μL, K_2_EDTA: 0.0109 ng/μL), with no significant difference on log‐transformed paired analysis (*P* = 0.26). evDNA concentrations showed the same pattern (ACD: 0.00811 ng/μL, K_2_EDTA: 0.00813 ng/μL; *P* = 0.79). Strong correlations between paired measurements (cfDNA: *r* = 0.78, *P* = 0.007; evDNA: *r* = 0.89, *P* = 0.0006) confirmed highly consistent recovery across tube types. These results demonstrate that, under standardized handling, ACD and K_2_EDTA tubes yield equivalent cfDNA and evDNA recovery, supporting their interchangeable use in clinical liquid biopsy workflows.

### 3.4 Volume counts: Increased plasma volume improves evDNA recovery

**Fig. 5:**
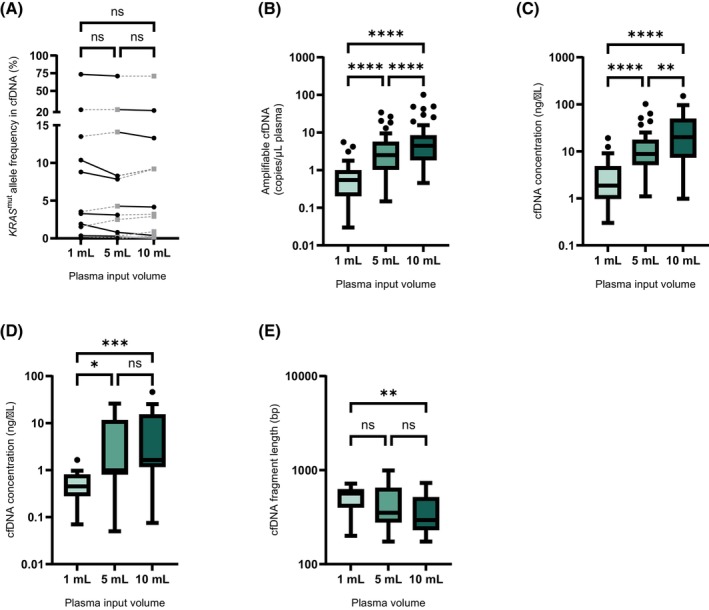
Impact of plasma input volume on cfDNA yield and fragment profiles. (A) *KRAS*‐mutant (*KRAS*ᵐᵘᵗ) allele frequency (%) in circulating cell‐free DNA (cfDNA) measured by droplet digital PCR (ddPCR) across plasma input volumes of 1, 5 mL, and 10 mL; gray dashed lines indicate increases; black solid lines indicate decreases in paired samples. (B) Amplifiable cfDNA (copies/μL plasma) determined by ddPCR for the same plasma input volumes (C) cfDNA concentration (ng/μL) measured by Qubit fluorometry. (D) cfDNA concentration (ng/μL) determined by TapeStation analysis. (E) cfDNA fragment length (bp) obtained by TapeStation analysis. Whole‐blood samples were processed within three hours of collection, and cfDNA was extracted from plasma input volumes of 1, 5, and 10 mL. Panels A is displayed on a linear scale, panels B–E on a log_10_ scale. Data are shown as box‐and‐whisker plots (Tukey method). Each data point represents an independent patient sample from the plasma input volume cfDNA cohort (*n* = 55). Statistical significance was assessed using the Friedman test followed by Dunn's multiple comparisons test (A), repeated‐measures one‐way ANOVA with Geisser–Greenhouse correction followed by Tukey's multiple comparisons test (B), and mixed‐effects models for repeated measures with Geisser–Greenhouse correction followed by Tukey's multiple comparisons test (C–E). ns = not significant, *P < 0.05, **P < 0.01, ***P < 0.001, ****P < 0.0001.

In this prospective cohort (*n* = 55), cfDNA was extracted from 1‐, 5‐, and 10‐mL plasma input volumes to evaluate the effect of sample volume on DNA yield and mutation detection. While ddPCR analysis showed comparable *KRAS* ^mut^ detection rates across all input volumes (Fig. 5A), an incremental trend was observed: five patients who were *KRAS* ^mut^‐negative at lower input volumes had detectable *KRAS* ^mut^ levels at 5 mL (*n* = 2) and 10 mL (*n* = 3), suggesting that higher plasma input may enhance mutation detection in borderline cases.

As expected, amplifiable genome equivalents increased significantly with increasing input volume, indicating improved ddPCR‐derived cfDNA recovery for downstream analysis (Fig. 5B). Qubit and TapeStation analyses confirmed a significant rise in cfDNA concentration with larger plasma input volumes (Fig. 5C,D), while TapeStation fragment analysis revealed an overall decrease in cfDNA fragment length with increasing input volume (Fig. 5E).

**Fig. 6:**
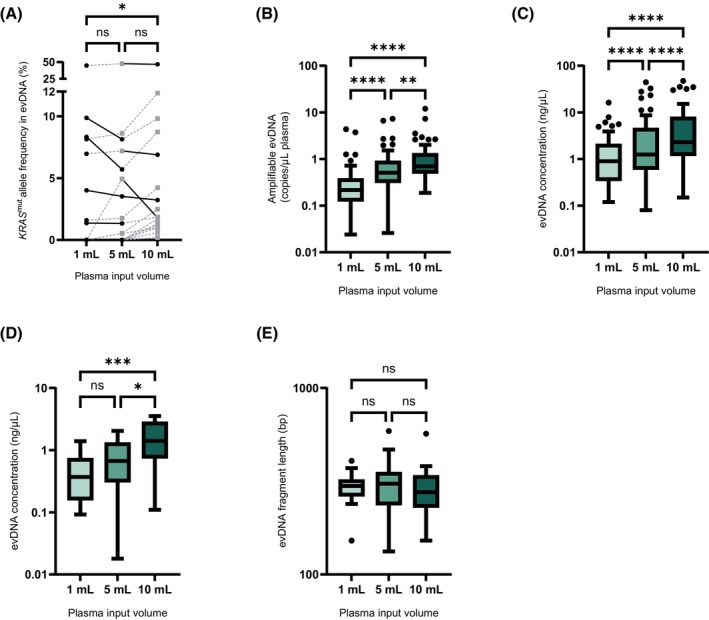
Impact of plasma input volume on evDNA yield and fragment profiles. (A) *KRAS*‐mutant (*KRAS*ᵐᵘᵗ) allele frequency (%) in extracellular vesicle‐derived DNA (evDNA) measured by droplet digital PCR (ddPCR) across plasma input volumes of 1, 5, and 10 mL; gray dashed lines indicate increases; black solid lines indicate decreases in paired samples. (B) Amplifiable evDNA (copies/μL plasma) determined by ddPCR for the same plasma input volumes. (C) evDNA concentration (ng/μL) measured by Qubit fluorometry. (D) evDNA concentration (ng/μL) determined by TapeStation analysis. (E) evDNA fragment length (bp) obtained by TapeStation analysis. Whole‐blood samples were processed within 3 hours of collection, and evDNA was extracted from plasma input volumes of 1, 5, and 10 mL. Panels A is displayed on a linear scale, panels B–E on a log_10_ scale. Data are shown as box‐and‐whisker plots (Tukey method). Each data point represents an independent patient sample from the plasma input volume evDNA cohort (*n* = 51). Statistical significance was assessed using the Friedman test followed by Dunn's multiple comparisons test (A, B), a mixed‐effects model for repeated measures with Geisser–Greenhouse correction followed by Tukey's multiple comparisons test (C), and repeated‐measures one‐way ANOVA with Geisser–Greenhouse correction followed by Tukey's multiple comparisons test (D, E). ns = not significant, *P < 0.05, **P < 0.01, ***P < 0.001, ****P < 0.0001.

We then evaluated evDNA performance (*n* = 51) by measuring *KRAS* ^mut^ in plasma input volumes of 1, 5, and 10 mL. ddPCR analysis showed an increase in *KRAS* ^mut^ detection between 1 and 10 mL plasma input volumes (Fig. 6A). Importantly, three samples became *KRAS* ^mut^‐positive at 5 mL and nine at 10 mL input, while they were negative at lower volumes.

Amplifiable genome equivalents also increased significantly, confirming enhanced evDNA recovery with greater input (Fig. 6B). Qubit and TapeStation analyses confirmed significant increases in evDNA concentration with rising volume (Fig. 6C,D), while TapeStation fragment analysis demonstrated stable evDNA fragment lengths (< 400 bp) across all volumes (Fig. 6E).

## 4 Discussion

Liquid biopsies are playing an increasingly critical role in clinical oncology, with numerous studies demonstrating the utility of cfDNA—and more recently, evDNA—as noninvasive biomarker for cancer detection, molecular profiling, and treatment monitoring. Their clinical significance is underscored by the growing number of liquid biopsy assays, granted clearance by the US Food and Drug Administration (FDA) in recent years [68, 69, 70, 71].

This evolving landscape supports a paradigm shift in which molecularly guided therapies may increasingly be prescribed solely on blood‐based testing, thereby eliminating the need for invasive tumor biopsies and enabling real‐time longitudinal monitoring throughout the treatment course. However, this potential hinges on the analytical robustness and reproducibility of liquid biopsy assays, both of which remain vulnerable to pre‐analytical variability. The most pressing challenge to clinical implementation is the lack of harmonized workflows [35, 38]. As liquid biopsy technologies continue to evolve, rigorous standardization becomes even more critical.

These challenges underscore that standardization of pre‐analytical variables is not only necessary for laboratory reproducibility, it is critical to ensure that patients receive accurate diagnoses and appropriate therapies. Several leading organizations have issued best‐practice recommendations, including the Association for Molecular Pathology (AMP) [72], the European Society for Medical Oncology (ESMO) [73], the American Society of Clinical Oncology and College of American Pathologists (ASCO/CAP) [74], the International Standards Organization (ISO) [75] and the US National Cancer Institute (NCI) [38]. Nevertheless, high‐quality, evidence‐based clinical guidelines remain limited [68, 76], underscoring the need for robust, empirically validated protocols.

In this study, we systematically evaluated key pre‐analytical variables that are readily modifiable in clinical and research workflows and their impact on cfDNA and evDNA quality using complementary analytical techniques.

While most previous investigations have focused on cfDNA, our study provides a direct comparison of pre‐analytical effects on both cfDNA and evDNA. This is especially relevant given the increasing interest in evDNA as a complementary analyte, offering potentially greater stability. Nonetheless, logistic and resource requirements such as the need for ultracentrifugation—the current gold standard—or alternative isolation methods [77], extended processing times, and higher costs must be weighed against its potential diagnostic advantages. EV isolation and downstream analysis remain labor‐intensive and require specialized equipment, while standardized normalization and manufacturing protocols are still lacking, increasing variability and limiting scalability [78, 79]. These logistic and methodological barriers currently confine evDNA to research applications, whereas cfDNA remains the more feasible analyte for routine clinical implementation.

Our data, drawn from one of the largest combined prospective and retrospective cohorts, demonstrate that delayed blood processing compromises analytical performance, particularly for mutation detection. In the prospective cohort, cfDNA concentrations increased significantly after 24 h and evDNA after 48 h at room temperature, whereas the larger retrospective cohort revealed earlier changes, with cfDNA rising after 6 h and evDNA after 24 h.

These findings supports the hypothesis that EV offer partial protection, preserving cargo integrity under suboptimal handling conditions [42, 43].

The earlier onset observed in the retrospective cohort likely reflects its greater statistical power and biological heterogeneity, enabling detection of degradation effects not visible in smaller, tightly controlled datasets. Our results align with previous reports recommending a processing window of ≤ 6 h for cfDNA [54, 72, 80] and extend these observations by providing quantitative thresholds for evDNA. As storage time is highly dependent on the tube type, our results specifically apply to ACD tubes, whereas cell‐stabilizing tubes have been shown to preserve cfDNA for a longer time [54].

Our findings confirm that storage temperature is a critical pre‐analytical variable influencing cfDNA and evDNA yield as well as mutation detection [43, 45]. Plasma cryopreservation at −80 °C substantially reduced cfDNA concentration and *KRA*S^mut^ detection, while evDNA concentration also decreased but mutation detection remained comparatively stable. Importantly, storage of already‐extracted DNA at −80 °C did not affect quantification or mutation detection, underscoring that degradation is primarily driven by plasma freezing rather than storage of purified DNA.

Short‐term refrigeration at 4 °C emerged as the most suitable alternative when immediate processing is not feasible. Although cfDNA yield decreased slightly compared to freshly processed samples, 4 °C consistently preserved cfDNA quality and amplifiable genome equivalents more effectively than −80 °C storage. For evDNA, 4 °C had minimal impact on yield or *KRA*S^mut^ detection, with significant reductions observed only after −80 °C storage, further underscoring the greater robustness of EV‐derived nucleic acids. Interestingly, our results diverge from those of El Messaoudi *et al*. [48], who reported no effect of short‐term storage across temperatures. However, their study evaluated only a 3‐h interval, whereas our extended storage periods revealed temperature‐dependent degradation, particularly for cfDNA.

Our results align with prior studies advocating 4 °C as a viable short‐term condition for cfDNA [45, 46, 81, 82] and evDNA [43], and reinforce evidence that evDNA better withstands temperature fluctuations [42].

Taken together, our data support current best‐practice recommendations favoring prompt processing of plasma samples and highlight 4 °C storage as a superior short‐term option when immediate extraction is not feasible. Long‐term preservation is best achieved by extracting DNA first, followed by cryopreservation.

The choice of blood collection tube is a practical yet often overlooked pre‐analytical variable. Several studies have evaluated alternative tubes containing cell‐stabilizing preservatives [34, 36, 83, 84], and one study comparing Streck tubes with K_2_EDTA found no significant difference in allele frequencies for NGS‐based mutation detection [51]. While stabilizing tubes offer extended processing windows, their higher cost and limited availability reduce feasibility for large‐scale, multi‐institutional clinical trials.

In our study, cfDNA and evDNA yields were equivalent between ACD and K_2_EDTA tubes when processed within 3 hours. These findings are consistent with current best practices recommending K_2_EDTA tubes when prompt processing (under 6 hours) is ensured [38, 74, 85].

Although Sato *et al*. [82] reported benefit of citrate‐based tubes under certain conditions, our data support the interchangeable use of both tube types in controlled workflows.

Plasma input volume remains an underexplored pre‐analytical factor, and no standardized recommendations currently exist to guide optimal volume selection [37]. Prior reports have suggested that increased input may enhance cfDNA recovery and improve mutation detection [59].

In our study, cfDNA yield increased with larger input volumes, but *KRAS* ^mut^ detection rates by ddPCR did not significantly improve, suggesting that 1 mL of plasma is often sufficient for highly sensitive assays. In contrast, evDNA yield and *KRAS* ^mut^ detection improved markedly with higher volumes, with several samples converting from negative to positive at 5 mL or 10 mL. These results support the notion that EV carry a tumor‐enriched DNA fraction and indicate that ≥ 5 mL input is advantageous for evDNA‐based workflows.

Despite the comprehensive design, this study has several limitations. Subgroup analyses with smaller sample sizes, such as *KRAS* detection in K_2_EDTA tubes, have reduced statistical power. While ddPCR provides ultra‐sensitive mutation detection, findings may not directly extrapolate to NGS without cross‐platform validation. The predominance of PDAC samples may limit generalizability across tumor types.

Additionally, the analytical platforms used, ddPCR, Qubit, and TapeStation, measure distinct molecular properties that are not directly interchangeable. However, the consistent directional trends support the robustness of our conclusions.

Finally, *KRAS* was selected as the molecular readout due to its high prevalence and clinical relevance in PDAC and CRC. While this choice enables focused validation, broader genomic panels will be necessary in future studies to assess whether the pre‐analytical trends observed here extend to other clinically actionable mutations across tumor types.

The results of this study have direct implications for optimizing liquid biopsy workflows. Pre‐analytical variability remains a major contributor to assay inconsistency, particularly when processing occurs outside centralized laboratories. To translate these findings into actionable practice, we propose the following best‐practice recommendations to improve standardization and analytical performance of cfDNA and evDNA‐based liquid biopsy assays:

- Processing time: Process ACD blood samples within 6 h for cfDNA and within 24 h for evDNA.
- Storage conditions: Process plasma immediately. When immediate extraction is not feasible, store plasma at 4 °C; for long‐term preservation, extract DNA prior to freezing at −80 °C.
- Tube type: Both ACD and K_2_EDTA tubes are suitable when processing occurs within recommended timeframes.
- Plasma input volume: Use ≥ 1 mL plasma for cfDNA analysis and ≥ 5 mL for evDNA to optimize DNA yield and detection sensitivity.

## 5 Conclusions

This study highlights the substantial impact of pre‐analytical variables on the quality and detectability of cfDNA and evDNA in liquid biopsy workflows. By systematically analyzing processing time, storage temperature, plasma input volume, and blood collection tubes, we provide practical, evidence‐based recommendations to improve assay consistency and clinical reliability.

Standardizing pre‐analytical protocols is essential for maximizing diagnostic yield, minimizing false negatives, and enabling reproducible results across institutions. While our findings focused on cfDNA and evDNA, these principles likely extend to other analytes such as methylated DNA, microRNA, and exosomal proteins.

As liquid biopsy technologies advance, harmonized workflows will be critical for expanding their role in early cancer detection, treatment stratification, and longitudinal monitoring. Broader adoption in routine care and clinical trials will depend not only on molecular innovation, but on the rigor of foundational protocols that ensure reliable and clinically meaningful data.

## Conflict of interest

AM is listed as an inventor on a patent that has been licensed by Johns Hopkins University to Thrive Earlier Detection and serves as a consultant for Tezcat Biosciences. The remaining authors declare no conflicts of interests.

## Author contributions

AM, PAG, AS were involved in conceptualization. JD, LS, BS, PAG, AS were involved in methodology. JD, VB, AM, PAG, AS were involved in validation. JD, BS were involved in formal analysis. JD, LS, BS, VB, LB, PL, JR, DW, AS were involved in investigation. VB, JCK, AM, PAG, AS were involved in resources. JD, LS, BS were involved in data curation. JD, LS, BS were involved in writing—original draft. JD, LS, VB, LB, PL JR, DW, PAG, AS were involved in writing—review and editing. JD was involved in visualization. JCK, AM, PAG, AS were involved in supervision. AM, PAG, AS were involved in project administration and funding acquisition.

## Supporting information


**Fig. S1.** Stage‐stratified *KRAS*‐mutant allele frequencies in cfDNA from patients with pancreatic ductal adenocarcinoma (PDAC).

**Fig. S2.** Fragment profiles of cfDNA and evDNA following delayed processing.

**Fig. S3.** Effect of plasma cryopreservation on cfDNA and evDNA quantity and fragment profiles.

**Fig. S4.** Effect of post‐extraction cryopreservation on cfDNA and evDNA quantity and mutation detection.

**Fig. S5.** Effect of short‐time plasma storage temperature on cfDNA and evDNA yield.

**Fig. S6.** Effect of short‐term plasma storage temperature on cfDNA and evDNA yield in an expanded clinical cohort.

**Table S1.** Clinical Characteristics of patients in *Delayed processing time cohort* (*n* = 32).
**Table S2**. Clinical Characteristics of archived patient samples in our institutional liquid biopsy database (*n* = 723).
**Table S3**. Clinical Characteristics of Patients in *Storage temperature cohort* (*n* = 65).
**Table S4**. Clinical Characteristics of Patients in *Collection tube cohort* (*n* = 12).
**Table S5**. Clinical Characteristics of Patients in the cfDNA *Plasma input volume cohort* (*n* = 55).
**Table S6**. Clinical Characteristics of Patients in the evDNA *Plasma input volume cohort* (*n* = 51).

## Data Availability

The datasets generated and analyzed during the current study are available from the corresponding author upon reasonable request. Due to patient confidentiality and institutional data protection policies, individual‐level clinical data are not publicly available.
