# Supplementary figures and images for: Pre‐analytical optimization of cell‐free DNA and extracellular vesicle‐derived DNA for mutation detection in liquid biopsies
Source: Mol Oncol. 2026 Feb 22;20(7):1762–79. doi: 10.1002/1878-0261.70222 (PMC13352965; doi:10.1002/1878-0261.70222)

$KRAS^{mut}$  allele frequency  
in cfDNA (%)

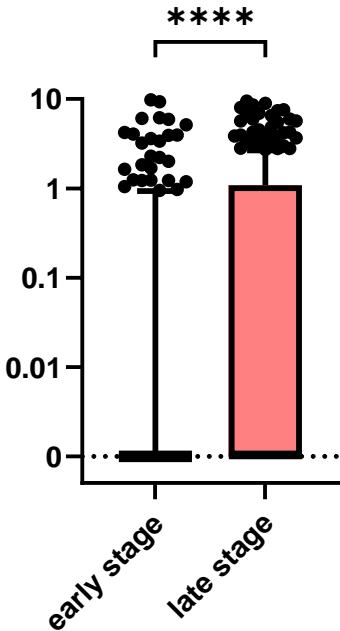

Supplement: Supplementary file 1 — Fig. S1. Stage‐stratified KRAS‐mutant allele frequencies in cfDNA from patients with pancreatic ductal adenocarcinoma (PDAC). [file MOL2-20-1762-s003.pdf]

**(A)**

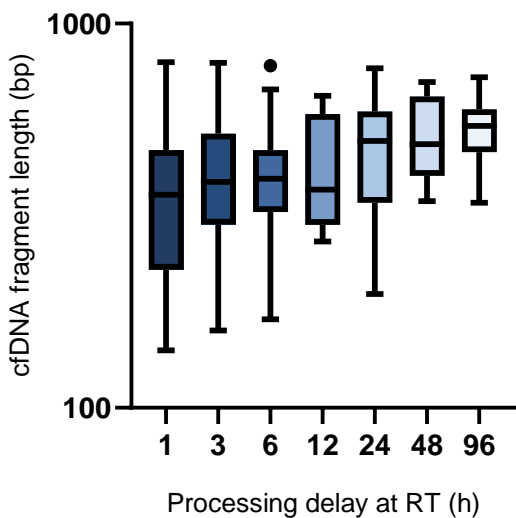

**(B)**

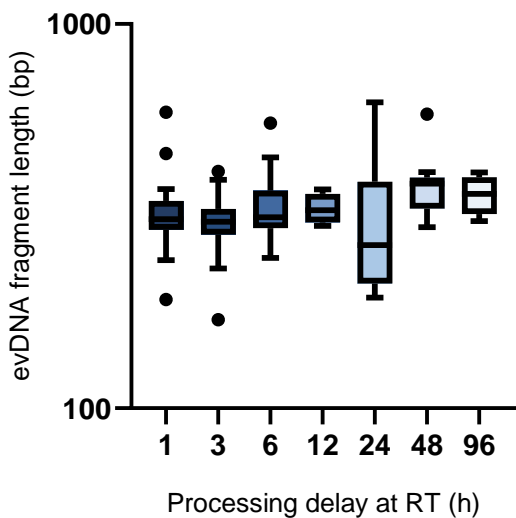

Supplement: Supplementary file 2 — Fig. S2. Fragment profiles of cfDNA and evDNA following delayed processing. [file MOL2-20-1762-s004.pdf]

**(A)**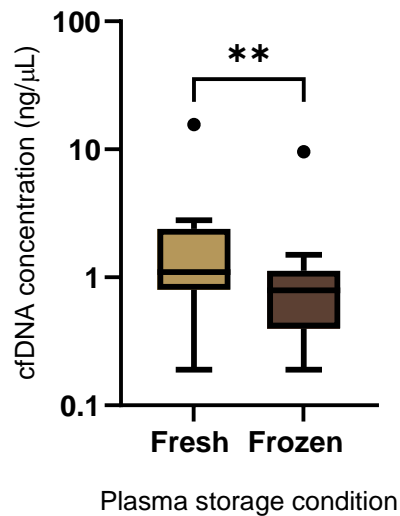**(B)**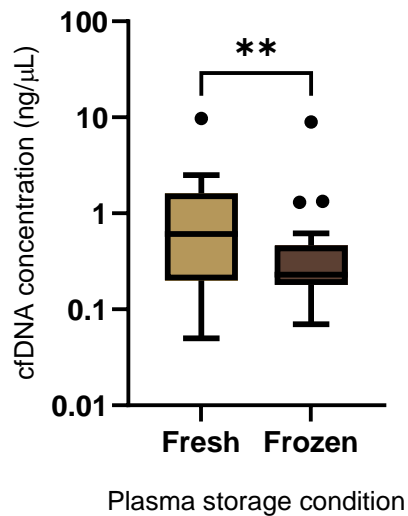**(C)**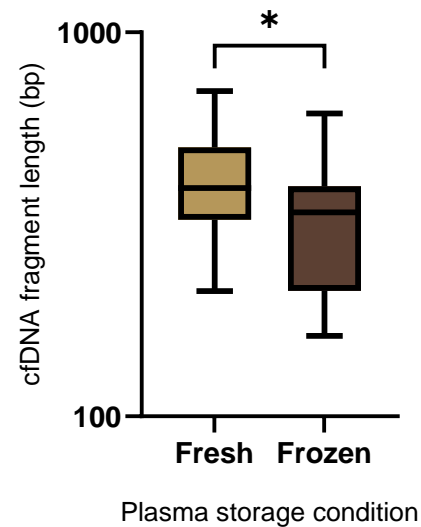**(D)**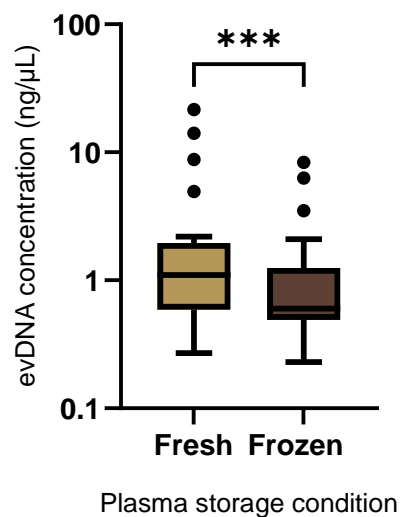**(E)**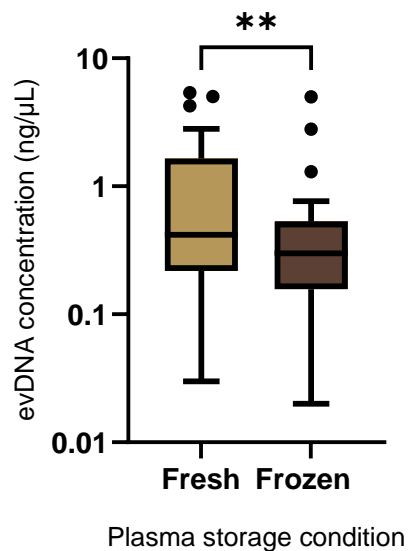**(F)**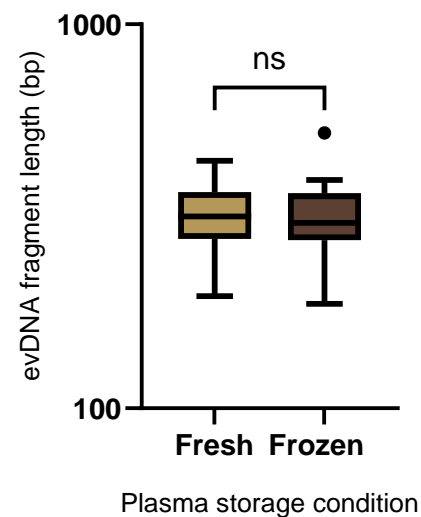

Supplement: Supplementary file 3 — Fig. S3. Effect of plasma cryopreservation on cfDNA and evDNA quantity and fragment profiles. [file MOL2-20-1762-s007.pdf]

**(A)**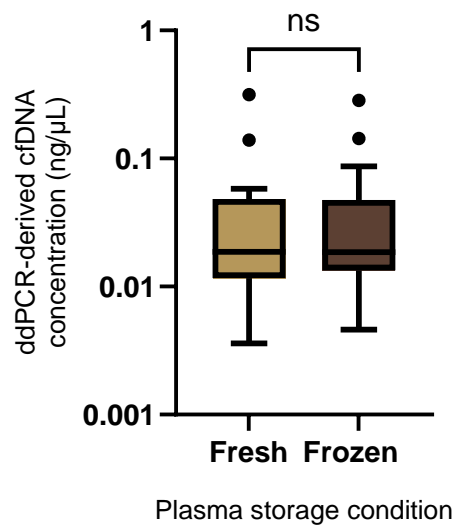**(B)**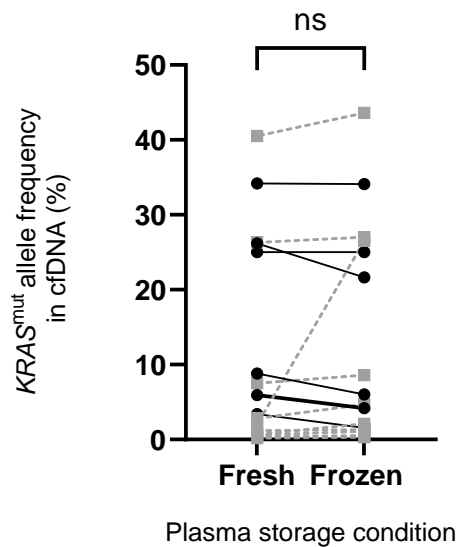**(C)**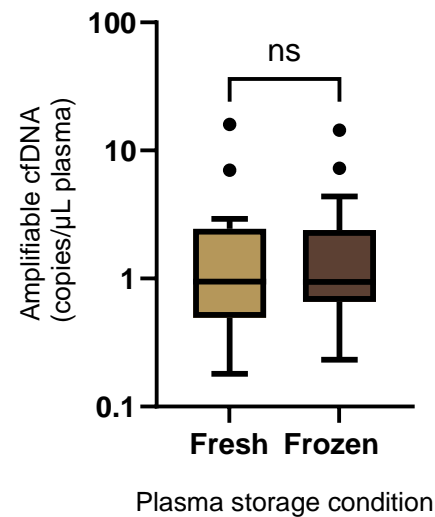**(D)**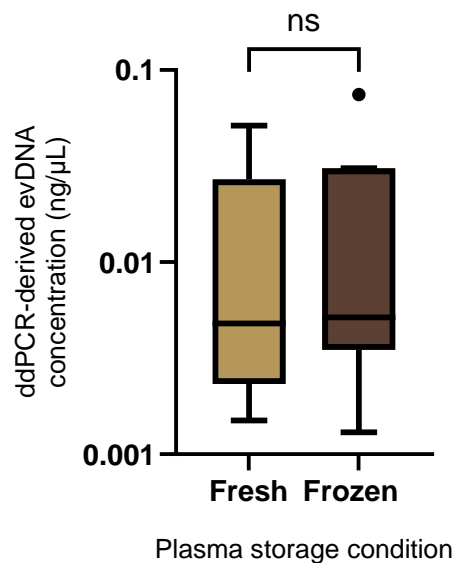**(E)**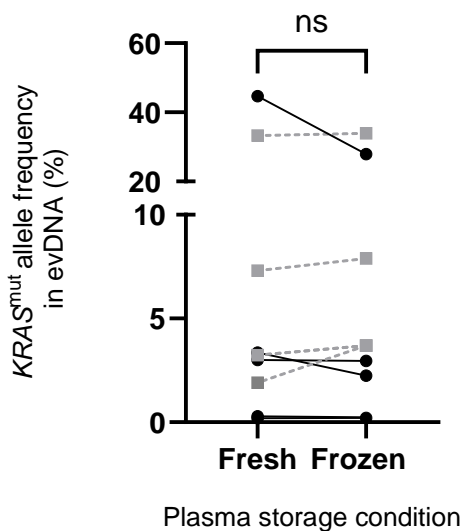**(F)**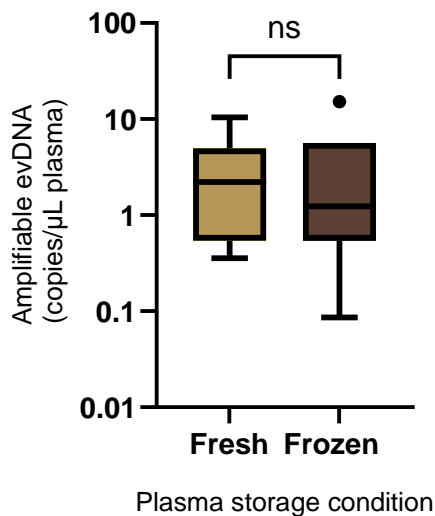

Supplement: Supplementary file 4 — Fig. S4. Effect of post‐extraction cryopreservation on cfDNA and evDNA quantity and mutation detection. [file MOL2-20-1762-s001.pdf]

**(A)**

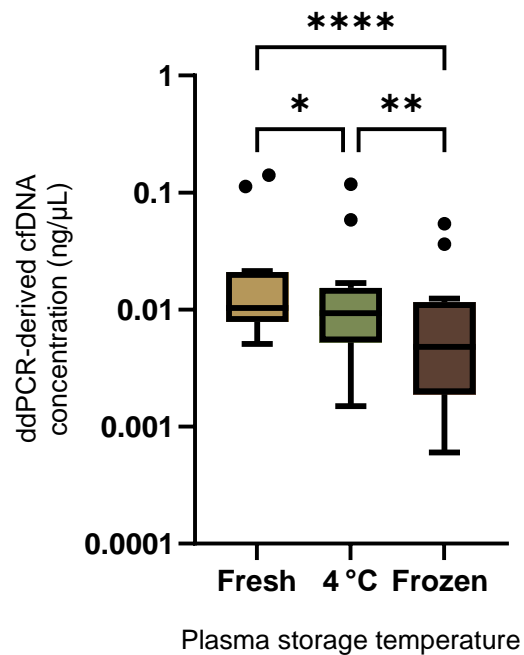

**(B)**

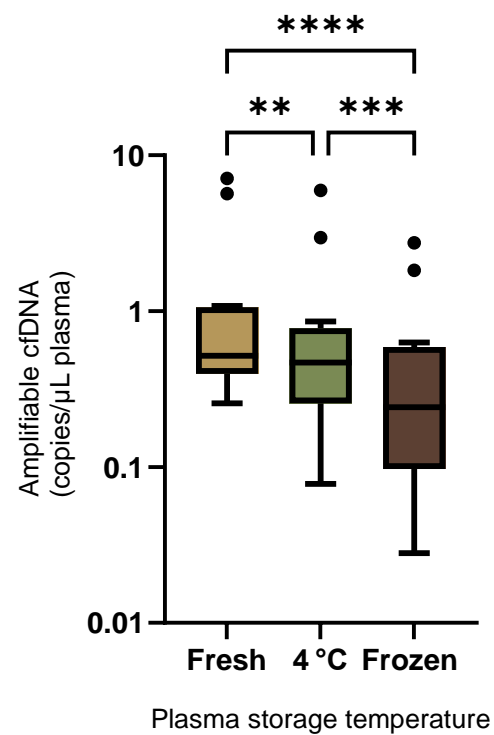

**(C)**

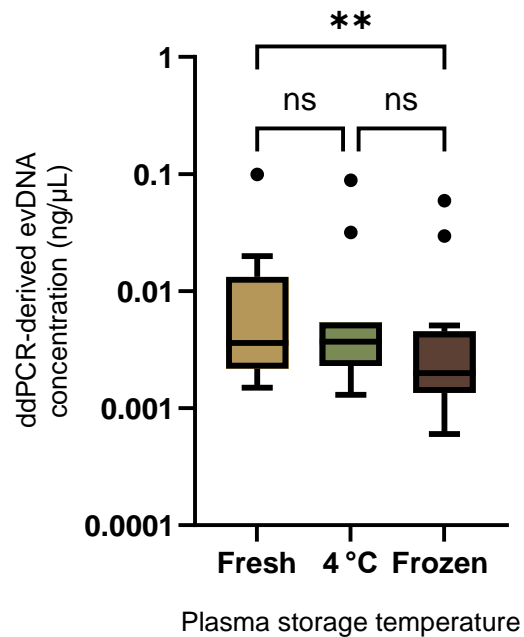

**(D)**

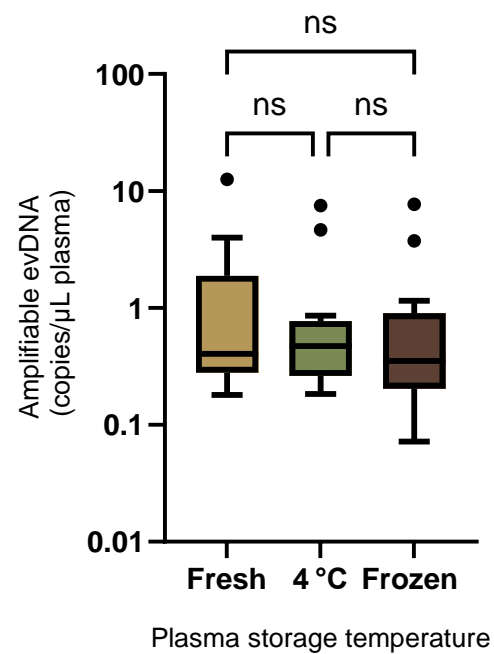

Supplement: Supplementary file 5 — Fig. S5. Effect of short‐time plasma storage temperature on cfDNA and evDNA yield. [file MOL2-20-1762-s002.pdf]

**(A)**

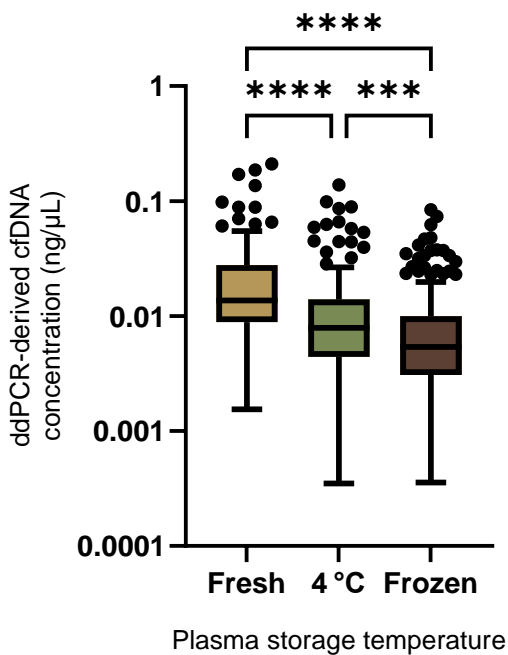

**(B)**

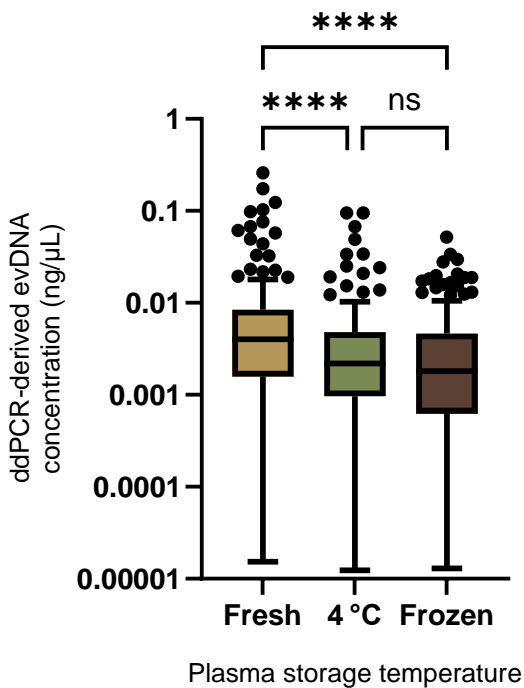

Supplement: Supplementary file 6 — Fig. S6. Effect of short‐term plasma storage temperature on cfDNA and evDNA yield in an expanded clinical cohort. [file MOL2-20-1762-s005.pdf]
