# Supplementary material for: Pre‐analytical optimization of cell‐free DNA and extracellular vesicle‐derived DNA for mutation detection in liquid biopsies
Source: Mol Oncol. 2026 Feb 22;20(7):1762–79. doi: 10.1002/1878-0261.70222 (PMC13352965; doi:10.1002/1878-0261.70222)
Supplement: Supplementary file 7 — Table S1. Clinical Characteristics of patients in Delayed processing time cohort (n = 32). Table S2. Clinical Characteristics of archived patient samples in our institutional liquid biopsy database (n = 723). Table S3. Clinical Characteristics of Patients in Storage temperature cohort (n = 65). Table S4. Clinical Characteristics of Patients in Collection tube cohort (n = 12). Table S5. Clinical Characteristics of Patients in the cfDNA Plasma input volume cohort (n = 55). Table S6. Clinical Characteristics of Patients in the evDNA Plasma input volume cohort (n = 51). [file MOL2-20-1762-s006.docx]

**Supplementary Tables**

**Supplementary Table S1:** Clinical Characteristics of patients in *Delayed processing time cohort* (n = 32)

| **Characteristic** | **Value** |
| --- | --- |
| **Total number of patients** | 32 |
| **Sex** | Male: 17 Female: 15 |
| **Age (years) ± Standard deviation (SD)** | Mean: 62 ± 11 Range: 37 – 83 |
| **Family history of cancer** | Yes: 15 No: 16 Not available: 1 |
| **Cancer type** | Pancreatic cancer (PDAC): 24 Colorectal cancer (CRC): 8 |
| **UICC tumor stage** (if applicable) | Stage I: 5 Stage II: 4 Stage III: 4 Stage IV: 16  Not available: 3 |

**Supplementary Table S2:** Clinical Characteristics of archived patient samples in our institutional liquid biopsy database (n = 723)

| **Characteristic** | **Value** |
| --- | --- |
| **Total number of patients** | 723 |
| **Sex** | Male: 389  Female: 334 |
| **Age (years) ± Standard deviation (SD)** | Mean: 63 ± 11 Range: 25 – 89 |
| **Family history of cancer** | Yes: 451 No: 172 Not available: 100 |
| **Cancer type** | Pancreatic cancer (PDAC): 641 Colorectal cancer (CRC): 82 |
| **UICC tumor stage** (if applicable) | Stage I: 91 Stage II: 143 Stage III: 92 Stage IV: 267  Not available: 130 |

**Supplementary Table S3.** Clinical Characteristics of Patients in *Storage temperature cohort* (n = 65)

| **Characteristic** | **Value** |
| --- | --- |
| **Total number of patients** | 65 |
| **Sex** | Male: 39 Female: 26 |
| **Age (years) ± Standard deviation (SD)** | Mean: 60 ± 11 Range: 27 – 85 |
| **Family history of cancer** | Yes: 46 No: 14 Not available: 5 |
| **Cancer type** | Pancreatic cancer (PDAC): 61 Colorectal cancer (CRC): 4 |
| **UICC tumor stage** (if applicable) | Stage I: 5 Stage II: 9 Stage III: 9 Stage IV: 40 Not available: 2 |

**Supplementary Table S4:** Clinical Characteristics of Patients in *Collection tube cohort* (n = 12)

| **Characteristic** | **Value** |
| --- | --- |
| **Total number of patients** | 12 |
| **Sex** | Male: 9  Female: 3 |
| **Age (years) ± Standard deviation (SD)** | Mean: 64 ± 12  Range: 47 – 77 |
| **Family history of cancer** | Yes: 8  No: 3  Not available: 1 |
| **Cancer type** | Pancreatic cancer (PDAC): 12 |
| **UICC tumor stage** | Stage I: 0  Stage II: 3  Stage III: 2  Stage IV: 7 |

**Supplementary Table S5:** Clinical Characteristics of Patients in the cfDNA *Plasma input volume cohort* (n = 55)

| **Characteristic** | **Value** |
| --- | --- |
| **Total number of patients** | 55 |
| **Sex** | Male: 29 Female: 26 |
| **Age (years) ± Standard deviation (SD)** | Mean: 64 ± 11 Range: 27 – 83 |
| **Family history of cancer** | Yes: 37 No: 13 Not available: 5 |
| **Cancer type** | Pancreatic cancer (PDAC): 48 Colorectal cancer (CRC): 7 |
| **UICC tumor stage** (if applicable) | Stage I: 12 Stage II: 13 Stage III: 8 Stage IV: 17 Not available: 5 |

**Supplementary Table S6:** Clinical Characteristics of Patients in the evDNA *Plasma input volume cohort* (n = 51)

| **Characteristic** | **Value** |
| --- | --- |
| **Total number of patients** | 51 |
| **Sex** | Male: 28 Female: 23 |
| **Age (years) ± Standard deviation (SD)** | Mean: 64 ± 11 Range: 33 – 85 |
| **Family history of cancer** | Yes: 32 No: 15 Not available: 4 |
| **Cancer type** | Pancreatic cancer (PDAC): 43 Colorectal cancer (CRC): 8 |
| **UICC tumor stage** (if applicable) | Stage I: 6 Stage II: 7 Stage III: 9 Stage IV: 22 Not available: 7 |

**Supplementary Figures**

**Supplementary Figure S1: Stage-stratified *KRAS*-mutant allele frequencies in cfDNA from patients with** **pancreatic ductal adenocarcinoma (PDAC)**

Box-and-whisker plots (Tukey method) show droplet digital PCR (ddPCR)-derived *KRAS*-mutant (*KRAS*ᵐᵘᵗ) allele frequencies (%) in circulating cell-free DNA (cfDNA) from patients with pancreatic ductal adenocarcinoma (PDAC), stratified by tumor stage according to the Union for International Cancer Control (UICC): early stage (UICC I/II; n = 189) and late stage (UICC III/IV; n = 337). The y-axis is displayed on a log₁₀ scale. Values above 10% were truncated for graphical clarity. Statistical significance was assessed using the Mann–Whitney U test. **** = p < 0.0001.

**Supplementary Figure S2: Fragment profiles of cfDNA and evDNA following delayed processing**

**(A)** Fragment size distribution (base pairs, bp) of circulating cell-free DNA (cfDNA) determined by TapeStation analysis after defined delays in whole-blood processing at room temperature (RT, 19–25 °C).

**(B)** Fragment size distribution (base pairs, bp) of extracellular vesicle-derived DNA (evDNA) determined under the same conditions**.**

*Whole-blood samples were stored at RT for 1–96 hours (h) prior to plasma separation (“processing delay”). Data are shown as box-and-whisker plots (Tukey method) on a log₁₀ scale. Each data point represents an independent patient sample from the delayed processing time cohort (n = 32 patients). Gradual colour shading (dark to light) indicates increasing processing delay. Statistical significance was assessed using a mixed-effects model for repeated measures, followed by Dunnett’s multiple comparisons test, using the 1 h time point as reference. No statistically significant differences in fragment size were observed for either cfDNA or evDNA across the investigated time points.*

**Supplementary Figure S3: Effect of plasma cryopreservation on cfDNA and evDNA quantity and fragment profiles**

**(A)** Circulating cell-free DNA (cfDNA) concentration (ng/µL) measured by Qubit fluorometry in freshly processed and cryopreserved (−80 °C) plasma.

**(B)** cfDNA concentration (ng/µL) determined by TapeStation analysis under identical conditions.

**(C)** cfDNA fragment length (base pairs, bp) obtained by TapeStation analysis.

**(D)** Extracellular vesicle–derived DNA (evDNA) concentration (ng/µL) measured by Qubit fluorometry in fresh and frozen plasma.

**(E**) evDNA concentration (ng/µL) determined by TapeStation analysis.

**(F)** evDNA fragment length (bp) measured by TapeStation analysis.

*Whole-blood samples were processed either immediately after collection (Fresh) or after plasma cryopreservation at −80 °C for two weeks (Frozen). Data are shown as box-and-whisker plots (Tukey method); concentration data are displayed on a log₁₀ scale. Each data point represents an independent patient sample from the long-term −80 °C storage temperature cohort (n = 65). Statistical significance was assessed using paired t-tests (A, C, E, F) and Wilcoxon matched-pairs signed-rank tests (B, D). ns = not significant, * = p < 0.05, ** = p < 0.01, *** = p < 0.001.*

**Supplementary Figure S4: Effect of post-extraction cryopreservation on cfDNA and evDNA quantity and mutation detection**

**(A**) Droplet digital PCR (ddPCR)-derived concentration of circulating cell-free DNA (cfDNA; ng/µL) measured immediately after extraction (Fresh) and after cryopreservation of purified DNA at −80 °C for two weeks (Frozen).

**(B)** *KRAS*-mutant (*KRAS*ᵐᵘᵗ) allele frequency (%) in cfDNA under identical conditions; grey dashed lines indicate increases; black solid lines indicate decreases in paired samples.

**(C)** Amplifiable cfDNA genome equivalents (copies/µL plasma) determined by ddPCR.

**(D)** ddPCR-derived extracellular vesicle-derived DNA (evDNA) concentration (ng/µL) measured immediately after extraction and after cryopreservation at −80 °C for two weeks.

**(E)** *KRAS*ᵐᵘᵗ allele frequency (%) in evDNA; grey dashed lines indicate increases; black solid lines indicate decreases in paired samples.

**(F)** Amplifiable evDNA genome equivalents (copies/µL plasma) determined by ddPCR.

*Purified cfDNA and evDNA samples were analyzed immediately after extraction (Fresh) or following cryopreservation at −80 °C for two weeks (Frozen). Panels A, C, D and F are displayed on a log₁₀ scale, panels B and E on a linear scale. Data are presented as box-and-whisker plots (Tukey method). Each data point represents an individual patient sample from the extracted DNA −80°C storage temperature cohort (n = 16). Statistical significance was assessed using paired t-tests for all panels. ns = not significant.*

**Supplementary Figure S5: Effect of short-time plasma storage temperature on cfDNA and evDNA yield**

**(A)** Droplet digital PCR (ddPCR)-derived concentration of circulating cell-free DNA (cfDNA; ng/µL) measured in freshly processed plasma (Fresh), after overnight storage at 4 °C, and after overnight cryopreservation at −80 °C (Frozen).

**(B)** Amplifiable cfDNA genome equivalents (copies/µL plasma) determined by ddPCR under identical conditions.

**(C)** ddPCR-derived concentration of extracellular vesicle-derived DNA (evDNA; ng/µL) measured in plasma aliquots stored under the same conditions.

**(D)** Amplifiable evDNA genome equivalents (copies/µL plasma) determined by ddPCR.

*Whole-blood samples were processed within three hours of collection, and plasma aliquots were either analyzed immediately (Fresh), stored overnight at 4 °C, or cryopreserved overnight at −80 °C (Frozen). Data are shown as box-and-whisker plots (Tukey method) on a log₁₀ scale. Each data point represents an individual patient sample from the short-term 4 °C vs. −80 °C storage temperature cohort (n = 13). Statistical significance was assessed using a repeated-measures one-way ANOVA with Geisser–Greenhouse correction followed by Tukey’s multiple comparisons test (A, B), and the Friedman test followed by Dunn’s multiple comparisons test (C, D). ns = not significant, * = p < 0.05, ** = p < 0.01, *** = p < 0.001, **** = p < 0.0001.*

**Supplementary Figure S6. Effect of short-term plasma storage temperature on cfDNA and evDNA yield in an expanded clinical cohort**

**(A)** Droplet digital PCR (ddPCR)-derived concentration of circulating cell-free DNA (cfDNA; ng/µL) measured in freshly processed plasma (Fresh), plasma stored overnight at 4 °C, and plasma stored overnight at −80 °C (Frozen).

**(B)** ddPCR-derived concentration of extracellular vesicle–derived DNA (evDNA; ng/µL) measured under identical storage conditions.

*Whole-blood samples were processed within three hours of collection (Fresh; n = 175, prospective cohort) or plasma samples from an institutional liquid biopsy database were analyzed after overnight storage at 4 °C (n = 176) or −80 °C (n = 248) (retrospective validation cohort). Data are shown as box-and-whisker plots (Tukey method) on a log_10_ scale.* *Statistical significance was assessed using an ordinary one-way ANOVA on log-transformed data, followed by Tukey’s multiple comparisons test. ns = not significant, *** = p < 0.001, **** = p < 0.0001*
